# Algorithmic jingle jungle: A comparison of implementations of principal axis factoring and promax rotation in R and SPSS

**DOI:** 10.3758/s13428-021-01581-x

**Published:** 2021-06-07

**Authors:** Silvia Grieder, Markus D. Steiner

**Affiliations:** 1 Division of Developmental and Personality Psychology, Department of Psychology, University of Basel, Missionsstrasse 62, 4055 Basel, Switzerland; 2 Center for Cognitive and Decision Sciences, Department of Psychology, University of Basel, Basel, Switzerland

**Keywords:** Software comparison, Exploratory factor analysis, Principal axis factoring, Promax rotation

## Abstract

A statistical procedure is assumed to produce comparable results across programs. Using the case of an exploratory factor analysis procedure—principal axis factoring (PAF) and promax rotation—we show that this assumption is not always justified. Procedures with equal names are sometimes implemented differently across programs: a jingle fallacy. Focusing on two popular statistical analysis programs, we indeed discovered a jingle jungle for the above procedure: Both PAF and promax rotation are implemented differently in the *psych* R package and in SPSS. Based on analyses with 247 real and 216,000 simulated data sets implementing 108 different data structures, we show that these differences in implementations can result in fairly different factor solutions for a variety of different data structures. Differences in the solutions for real data sets ranged from negligible to very large, with 42% displaying at least one different indicator-to-factor correspondence. A simulation study revealed systematic differences in accuracies between different implementations, and large variation between data structures, with small numbers of indicators per factor, high factor intercorrelations, and weak factors resulting in the lowest accuracies. Moreover, although there was no single combination of settings that was superior for all data structures, we identified implementations of PAF and promax that maximize performance on average. We recommend researchers to use these implementations as best way through the jungle, discuss model averaging as a potential alternative, and highlight the importance of adhering to best practices of scale construction.

Psychological research is mainly conducted using quantitative methods. Whereas in the early days of psychology statistical procedures had to be implemented by hand, today a variety of programs exists for this purpose. Generally, implementations of statistical procedures are thought to produce equivalent results across programs; at least their interchangeable use in scientific publications suggests as much. However, to date, detailed comparisons of these implementations (i.e., on a code or output level) are scarce (for exceptions, see, e.g., Stanley, 2015; Kotenko, 2017; Hodges, Stone, Johnson, Carter, & Lindsey, 2020), and it is thus unclear whether this interchangeable use of programs is justified (for meta-level comparisons, see, e.g., MacCallum, 1983; Marr-Lyon, Gupchup, & Anderson, 2012; Gosh, 2019; Klinke, Mihoci, & Härdle, 2010).

Our attention was drawn to this topic in the course of the review process of Grieder and Grob (2020), where the authors conducted exploratory factor analyses (EFA). A reviewer of Grieder and Grob (2020) suggested testing the robustness of results using a second program. It became apparent that although the same statistical methods were applied, results were not comparable between these programs, and even led to different interpretations and conclusions. As it turned out, this case was no exception (e.g., GaryStats, 2017; krissen, 2018; u/kriesniem, 2018; del Rio, 2017; Collins, 2016; Hodges et al., 2020, see also Newsom, 2020 on EFA). As Ershova and Schneider (2018) point out, results from a specific statistical method can even differ between different versions of the same program if the implementation of algorithms are changed, which often happens without explicit notification of the users.^1^ Given the instances referenced above, it seems likely that most people assume implementations of the same method in different programs to yield equivalent—or at least highly comparable—results. As a consequence, a researcher reporting results based on one program might be criticized if these results are not reproducible by another researcher employing another program. This might become more of an issue with increasing popularity and promotion of the open science movement (see Gernsbacher, 2018), as data may be shared more often, and it might also contribute to one of the most pressing contemporary issues in psychology—the replication crisis (Ershova & Schneider, 2018; Open Science Collaboration, 2015; Munafò et al., 2017), for example when it comes to ongoing construct validations (see Flake, Pek, & Hehman, 2017) or direct replication studies (Hodges et al., 2020). In factor analysis, the worst consequence of differences in implementations could be a misalignment of which indicator was classified to be part of which latent construct across results obtained from different implementations. This is exactly what happened in the personal example mentioned above, and it might seemingly yield evidence against the validity of a scale in ongoing scale validation (Flake et al., 2017), even though the differences might be due to the implementation of the same statistical procedure used.

The case where different concepts (in our case: different implementations of a statistical procedure) are referred to by the same name is known as the jingle fallacy (Thorndike, 1904). The present work provides a first step to gauging the extent to which a “jingle jungle” exists in the implementations of some statistical procedures across different programs. Moreover, we map this jungle by revealing different ways through it and gauge the resulting implications by looking at the size of possible differences in results. Finally, we try to navigate through the jungle by identifying the implementation that renders the most accurate results. In the present study, we focus on implementations of a specific procedure within a frequently used statistical framework—EFA—in two of the most often used programs for statistical analyses in psychological research (Dunn, 2011): *SPSS* (IBM Corp, 2020) and *R* (R Core Team, 2020).

## Exploratory factor analysis

Factor analysis is a widely used tool to identify latent constructs underlying task performance or responses to questionnaire items. In factor analysis, the variance in a larger number of variables or indicators is sought to be accounted for by a smaller number of latent factors. A data-driven approach to factor analysis is EFA, which was originally developed by Spearman (1904, 1927) as a method to extract a common factor—a mathematical entity that accounts for the interrelations of test scores from different cognitive tasks (i.e., for the positive manifold of cognitive performances). This common entity, the general factor, is the construct thought to underlie manifest variables, such as subtest scores from intelligence tests. In EFA, intercorrelations between a given set of indicators are analyzed and a smaller number of factors is extracted that explain a maximum of the common variance between these indicators.

Two crucial decisions have to be made in advance when performing an EFA. First, the number of factors to retain needs to be determined. It is recommended to use multiple retention criteria for this purpose. Auerswald and Moshagen (2019), for example, suggested using sequential *χ*^2^ model tests in combination with either parallel analysis (Horn, 1965), the empirical Kaiser criterion (Braeken & Van Assen, 2017), or the Hull method (Lorenzo-Seva, Timmerman, & Kiers, 2011). In addition to quantitative criteria, qualitative criteria, such as theoretical considerations and the plausibility of the factor solution (Fabrigar, Wegener, MacCallum, & Strahan, 1999; Watkins, 2018; Hayton, Allen, & Scarpello, 2004), should also be considered.

Second, the factor extraction method and the rotation method used to seek *simple structure*—that is, a solution where each indicator loads substantially onto one, and only one, factor—need to be chosen. One of the most commonly used factor extraction methods is iterative principal axis factoring (PAF). Compared to another frequently used and recommended method, maximum likelihood estimation (ML), PAF has several advantages. First, it has no distributional assumptions, whereas ML requires the data to follow a multivariate normal distribution (e.g., Fabrigar et al., 1999). Second, it is more robust in the case of unequal factor loadings, few indicators per factor, and small sample sizes (De Winter & Dodou, 2012; Briggs & MacCallum, 2003). Finally, it is better able to recover weak factors (Briggs & MacCallum, 2003; De Winter & Dodou, 2012). This means that PAF is less likely to produce Heywood cases (that is, communalities^2^> 1) and non-convergence in the aforementioned data structures compared to ML (Fabrigar et al., 1999; De Winter & Dodou, 2012; Briggs & MacCallum, 2003). On the other hand, ML readily enables the computation of multiple fit indices and of significance tests for the factor loadings (e.g., Fabrigar et al., 1999). In our study, we focus on PAF as a factor extraction method.

Once the specified number of common factors is extracted, the solution is typically rotated in an attempt to obtain simple structure. Rotations can be categorized into orthogonal and oblique rotations. In an orthogonally rotated solution, the resulting factors are uncorrelated. The most popular orthogonal rotation method is varimax (Kaiser, 1958; Watkins, 2018). In most cases, however, the resulting factors are assumed to be at least somewhat correlated and thus an oblique rotation is more appropriate. Many oblique rotation procedures also start with an orthogonal rotation, but then the orthogonality constraints are lessened and the factors are allowed to correlate. In the case of correlated factors, this results in a solution that approaches simple structure even further than an orthogonal solution. If the factors are uncorrelated in reality, an oblique rotation will produce orthogonal factors as well. This is why oblique rotations are generally recommended over orthogonal rotations (Watkins, 2018; Fabrigar et al., 1999; Gorsuch, 1983). The most popular oblique rotation methods are promax (Hendrickson & White, 1964) and oblimin (Jennrich & Sampson, 1966; Watkins, 2018; Carroll, 1958). If an oblique factor solution contains substantial factor intercorrelations, this implies that a hierarchical structure is present (i.e., one or more constructs can be assumed that explain these intercorrelations). In this case, it is useful to make the hierarchical nature explicit by transforming the oblique solution accordingly, for example using the Schmid-Leiman transformation (Schmid & Leiman, 1957). In this article, we focus on oblique factor solutions after promax rotation, as this can be used with both hierarchical and nonhierarchical data structures. The implementations of PAF and promax rotation in R and SPSS are described below.

## Present study

The main aim of this study is to compare implementations and results of a commonly used EFA procedure—PAF and promax rotation—in R, version 4.0.3 (R Core Team, 2020) and in SPSS, version 27 (IBM Corp, 2020).^3^ In R, we used the *psych* package, version 2.0.12. (Revelle, 2020); henceforth referred to as R psych), a very popular and extensive R package that has also influenced EFA implementations in Python (Biggs, 2019) and has been recommended to be used with the R plugin in SPSS for some calculations (IBM Support, 2020). We first compared the implementations in R psych and SPSS on a code-level. As the source code for SPSS is not publicly available, we relied on the algorithms manual in which the formulas of the implementations are provided (IBM Corp, 2017). As a next step, we were interested to see how the identified differences between the two implementations would impact results. To this end, we compared results from the two implementations for a large set of real data. Finally, we wanted to see whether there is an implementation of PAF and promax rotation that renders more accurate results in general, or at least for certain data structures. To this end, we compared the ability to recover a set of true population models across all possible combinations of the considered settings for PAF and promax rotation—including the two combinations used in R psych and SPSS—in a simulation study based on a large set of population models (varying, e.g., factor intercorrelations and the number of indicators per factor). This also allowed us to determine whether the implementation or the data structure is more important for accurate results. Thus, our goal was to explore whether a jingle jungle indeed exists for the investigated procedure, try to map it, and seek the best way through it.

To facilitate comparisons, we first ran analyses in the programs mentioned above and then reproduced results from both programs using our own functions included in a dedicated R package—*EFAtools* (Steiner & Grieder, 2020). We then conducted all further analyses with our own functions that enable a flexible use of all combinations of settings needed for the simulation analyses and that are faster due to C++ implementations of the iterative procedures. Results on how well our functions reproduced the original implementations in R psych and SPSS are provided below. Supplemental Material (SM) to this study, as well as all analysis scripts and many of the data sets used for the real data analyses, are available at https://osf.io/a836q.

## Implementations in R psych and SPSS

### Principal axis factoring

PAF is a least squares fitting approach in EFA. It uses the variances and covariances of a given set of indicators to reduce dimensionality by extracting a prespecified number of factors such that they explain a maximum of the common variance in these indicators (often, a correlation matrix is used to this end). The standard way of performing PAF in R is with the fa function in the psych package, and in SPSS with the FACTOR algorithm. We now briefly describe the differences in PAF implementations in the two programs; these are also listed in Table 3. A more detailed description of PAF and its implementation in R psych and SPSS is included in the SM (Section 1).

In a first step, both in R psych and SPSS, the correlation matrix is tested for different properties. If the correlation matrix is not positive definite, R psych will perform smoothing to produce a highly similar positive definite matrix to proceed with. In contrast, SPSS will throw an error and abort if a non-positive definite matrix is entered.

In a next step, initial communalities are estimated and used to replace the diagonal of the correlation matrix. Several approaches exist for deriving initial estimates; three of them being (a) *unity* (i.e., each initial communality is set to one, thus the correlation matrix remains unchanged), (b) the *maximum absolute correlation* (MAC) of an indicator with any other indicator, and (c) the *squared multiple correlations* (SMCs; Gorsuch, 1983; Harman, 1976). It has been advocated to rely on SMCs as initial estimates (Guttman, 1956; Wrigley, 1957; Roff, 1936; Dwyer, 1939)—which is what both the R psych and SPSS implementations do by default. When SMCs are entered into the diagonal of the correlation matrix, it is often the case that the matrix is no longer positive semidefinite; that is, some of its eigenvalues are negative. The PAF procedure, however, involves taking the square root of the *m* largest eigenvalues and therefore cannot be executed if any of these *m* eigenvalues—where *m* is the number of factors to extract—are negative. When SMCs cannot be used, R psych suggests using unity as initial communality estimates, which may lead to convergence, but may also lead to inflated final communality estimates (Gorsuch, 1983). It has therefore been recommended to use MACs instead of unity when SMCs fail (Gorsuch, 1983)—which is what SPSS supposedly does (IBM Corp, 2017). However, using SMCs rarely fails in SPSS, as SPSS takes the absolute of the eigenvalues, thereby avoiding negative eigenvalues during the iterative PAF procedure (IBM Corp, 2017). In R psych, using SMCs will fail whenever any of the *m* largest eigenvalues are negative. Thus, R psych and SPSS deal differently with negative eigenvalues, which results in different cases where using SMCs will fail.

After the initial communality estimates have been determined, the final communalities are estimated in an iterative process (see SM Section 1 for details). This process is continued until an arbitrary convergence criterion is reached, which, by default, is 10^− 3^ for both R psych and SPSS. Others have suggested more strict criteria, such as 10^− 5^ (Mulaik, 2010) or 10^− 6^ (Briggs & MacCallum, 2003). When testing convergence—that is, testing whether the differences from one iteration to the next are small enough and thus the current solution is considered stable—SPSS tests against the maximum difference in any single communality estimate, whereas R psych tests against the difference in the sum of all communalities.

To summarize, we found three differences between the R psych and SPSS PAF implementations, namely not taking versus taking the absolute value of eigenvalues, using unity versus MACs as initial communality estimates if SMCs fail, and using different referents when testing convergence.

### Promax rotation

Once the specified number of factors is extracted, a rotation is typically performed to achieve an interpretable solution. Promax is a fast and efficient method for oblique factor rotation. In this procedure, a varimax rotation (usually preceded by Kaiser normalization; Kaiser, 1958) is performed first to obtain an orthogonal solution, which is then transformed into an oblique solution (Hendrickson & White, 1964). Here, we again only briefly introduce the differences in promax implementations (see Table 3 for a summary). A more detailed description of promax and its implementation in R psych and SPSS is included in the SM (Section 1).

In SPSS, promax with Kaiser normalization is implemented as a rotation method in the FACTOR algorithm. In R, there are at least two functions available to perform a promax: the promax function in the *stats* package (R Core Team, 2020) and the Promax function in the *psych* package, both enabling promax rotation with and without Kaiser normalization. For comparability with SPSS, we used the promax implementation with Kaiser normalization called in the R psych fa function.

As stated above, a varimax rotation is performed first in the promax procedure. This rotation is implemented differently in R psych and SPSS. In SPSS, the original varimax procedure from Kaiser (1958) is implemented (IBM Corp, 2017). However, the varimax criterion seems to be slightly different from the original one (see SM, Section 1 for the original version and the adapted version implemented in *EFAtools*). The varimax function called in R psych instead uses singular value decomposition for the rotation and the sum of the singular values as varimax criterion (see also Jennrich, 2001).

Between the implementations of the subsequent steps of promax, we found only one more difference. While R psych exactly follows the original promax procedure reported in Hendrickson and White (1964), SPSS deviates from the original procedure in that it performs a row normalization of the target matrix from the varimax solution in the first step of the promax procedure (see SM, Section 1 for details). Cureton (1976) provides some evidence for promax with row normalization to outperform unnormalized (i.e., original) promax. However, there exists no further evidence for or against row normalization. In most studies on promax, either both versions or the original, unnormalized version from Hendrickson and White (1964) were used (e.g., Tataryn, Wood, & Gorsuch, 1999; Jennrich, 2006; Lorenzo-Seva, 1999).

In promax rotation, the elements of the target matrix from the varimax rotation are raised to a power *k*. Initial evidence suggested that *k* = 4 leads to the most accurate results (Hendrickson & White, 1964; Cureton, 1976), and both R psych and SPSS use this value by default. In contrast, more recent evidence based on a large-scale Monte Carlo simulation study showed that *k* = 3 is preferable in most cases for unnormalized promax, and *k* = 2 is preferable for normalized promax (Tataryn et al., 1999).

To summarize, we found two differences between the R psych and SPSS promax implementations, namely the type of the varimax rotation (original versus singular value decomposition) and the use of an unnormalized versus a row-normalized target matrix.

We thus indeed discovered a jingle jungle regarding the implementations of the same statistical procedure in two different programs and have begun to map it by pointing out the differences in the implementations. Next, we were interested in estimating the impact thereof on the resulting factor solutions. To be able to do so in the most efficient way, we reproduced the implementations with our own functions included in a dedicated R package (Steiner & Grieder, 2020).

### Reproduction with the EFAtools package

To facilitate comparisons between the two, we reproduced both the R psych and SPSS implementations in the *EFAtools* package in R (Steiner & Grieder, 2020). This enabled fast comparisons for multiple data sets in the same program.

We tested how well our EFA function was able to reproduce the R psych and SPSS implementations using real and simulated data sets. As real data sets, we used four correlation matrices also included in the real data analyses reported below. Specifically, these included data on the Domain-Specific Risk-Taking scale (DOSPERT), the Intelligence and Development Scales–2 (IDS-2), and the Woodcock-Johnson IV (WJIV) on 3- to 5- and 20- to 39-year-olds (see Table *Real_data_description.xlsx* in the online repository for descriptions, https://osf.io/pcrqu/). As simulated data sets, we used correlation matrices derived from four selected population models constructed for the simulation analyses reported below: Case 18|3|6, case 6|3|6, case 18|3|46|3c, and case 18|6|369wb, all with strong factor intercorrelations (see Table 4 for an overview of the cases, and SM, Sections 5 and 6 for the detailed models). For the pattern matrices of these population models, we apply the following naming convention throughout the manuscript: the population pattern matrices are indicated with a code in the form *p*|*m*|***λ***, where *p* is the number of indicators, *m* the true number of factors, and ***λ*** the set of unique non-zero pattern coefficients without the period (e.g., Case 18|6|369wb is a pattern matrix with 18 indicators, six factors, and non-zero pattern coefficients of .3, .6, and .9, mixed within and between factors). If cross-loadings are present, their number is indicated in a fourth compartment, as in case 18|3|46|3c, where 3 cross-loadings are present.

With the EFAtools package, we were able to reproduce all unrotated PAF loadings, varimax loadings, and pattern coefficients from a promax rotation from the R psych fa function to at least the 14th decimal (see Table S1 for detailed results). From the SPSS FACTOR algorithm, we were able to reproduce unrotated PAF loadings to at least the 9th decimal, and both varimax loadings and pattern coefficients from a promax rotation to at least the 4th decimal (see Table S1 for detailed results).

## Differences between the R psych and SPSS solutions for real data sets

Our previous analyses show that a jingle jungle does indeed exist for the implementations of PAF and promax in R psych and SPSS. But what impact does this have on the resulting factor solutions? As a first means to answer this question, we used correlation matrices from a heterogeneous collection of real data sets, mainly on cognitive abilities. These data sets varied with respect to the number of indicators, number of proposed first-order factors, and sample characteristics such as sample size, age, sex, socioeconomic status, and health status.

### Methods

#### Data sets

We analyzed a total of 247 correlation matrices, of which 219 were on cognitive abilities, 19 on personality, six on risk taking, and three on health and physical variables. Sample sizes varied between 22 and 619,150 (*Mdn*= 180), the number of indicators varied between 6 and 300 (*Mdn* = 16), the proposed number of first-order factors varied between 1 and 45 (*Mdn*= 4), the indicator-to-factor ratio varied between 2.0 and 34.0 (*Mdn* = 4.2), and the sample size-to-indicator ratio varied between 1.2 and 5,159.6 (*Mdn*= 9.3). For more information on the data sets and their sources, see Table *Real_data_description.xlsx* in the online repository (https://osf.io/pcrqu/).

#### Statistical analyses

As a first step, we determined the number of factors to extract. To this end, for each data set, we first determined the proposed number of factors from the literature and second, performed a parallel analysis based on SMCs with 1000 simulated data sets.^4^ Based on recommendations by Crawford et al., (2010), we tested the empirical eigenvalues of the data against the 95th percentile of the random eigenvalues for the first factor and against the mean random eigenvalues for subsequent factors.

As is commonly done, we then used the larger of the two numbers of factors—theoretical or data-driven—as an initial number of factors to extract for PAF with promax rotation. If no admissible solution was found with this initial number of factors after promax rotation, the number of factors was reduced by one and the procedure was repeated, and so on, until an admissible solution was achieved with both implementations (R psych and SPSS). A solution was deemed admissible if there were no Heywood cases (defined as communalities or unrotated loadings/structure coefficients ≥.998) and each factor displayed at least two salient pattern coefficients (with a threshold of ≥.20; Gorsuch, 1983; Kline, 1997).^5^

We recorded the number of factors for the final promax–rotated solution that worked for both implementations and the number of factors for the first admissible solution for both the R psych and SPSS implementations, to gauge how frequently these differed. Then, we determined the frequencies of differences in indicator-to-factor correspondences between the final solutions from R psych and SPSS. A different indicator-to-factor correspondence occurs if the same indicator loads saliently onto different factors in the two solutions, or if it only displays a salient loading in one solution, but not in the other. Hence, differences in indicator-to-factor correspondences are also possible for one-factor solutions.

Moreover, we examined overall, mean, and maximum absolute differences in loadings/pattern coefficients after PAF without rotation, PAF with varimax rotation, and PAF with promax rotation for each data set. By separating these three analysis steps, we were able to determine which led to the largest differences in results. In addition to differences in loadings, we also examined overall, mean, and maximum factor congruence for these three analysis steps. Factor congruence is an indicator of the similarity between factors that ranges from -1 to 1, with higher values indicating higher similarity (Burt, 1948). Values between .85 and .94 are interpreted as fair similarity, and values higher than .95 as good similarity (Lorenzo-Seva and Ten Berge, 2006).

Finally, we investigated a possible relationship of the mean and maximum average absolute differences in pattern coefficients from the final promax-rotated solutions with the indicator-to-factor ratio, as this ratio has been shown to affect the accuracy of a factor solution (MacCallum, Widaman, Zhang, & Hong, 1999). To achieve this, we performed Bayesian gamma regression analyses with a log-link function using the *rstanarm* R package (Goodrich, Gabry, Ali, & Brilleman, 2018) with default priors and the *bayestestR* package, version 0.7.2 (Makowski, Ben-Shachar, & Lüdecke, 2019). We determined credibility with the 95% highest density interval (HDI; Kruschke, 2018).

### Results

Of the 247 data sets subjected to PAF with promax rotation, 31 produced non-admissible solutions only and were therefore not included in further analyses. Of the 216 data sets for which final admissible solutions were found in both R psych and SPSS, the number of factors for the first admissible solution in R psych and SPSS differed in 5.1% of the data sets, with the solution from one implementation displaying up to four factors more than the solution from the other implementation. The final solutions on which all further comparisons between the two implementations were based were all achieved using SMCs as initial communalities (i.e., the *m* largest eigenvalues were positive for all solutions).

**Fig. 1:**
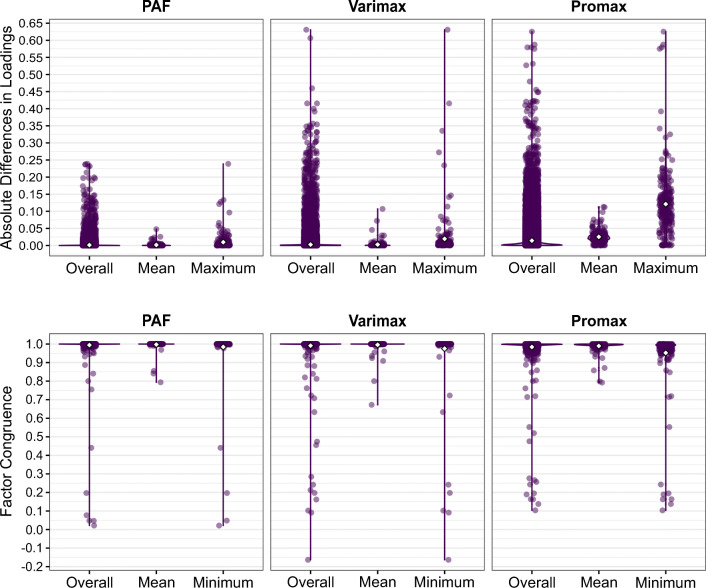
Overall, mean, and maximum absolute differences in loadings/pattern coefficients per solution and overall, mean, and minimum factor congruence per solution from PAF without rotation, with varimax rotation, and with promax rotation, obtained with the R psych and SPSS implementations for 216 real data sets. The *white diamond* represents the mean. PAF = principal axis factoring

Figure 1 shows the distributions of the overall, mean, and maximum absolute differences in loadings/pattern coefficients as well as the overall, mean, and minimum factor congruence between the R psych and SPSS solutions. The absolute differences in unrotated PAF loadings ranged overall between 0 and 0.24, the average mean absolute difference per solution (*M*_*mean*_) was 0.002, and the average maximum absolute difference per solution ($M_{\max \limits }$) was 0.01. After varimax rotation, the absolute differences in loadings ranged overall between 0 and 0.63 (*M*_*mean*_ = 0.003, $M_{\max \limits } = 0.02$), and after promax rotation, the absolute differences in pattern coefficients ranged overall between 0 and 0.63 (*M*_*mean*_ = 0.03, $M_{\max \limits } = 0.12$). The factor congruence for the unrotated PAF solutions ranged overall from .02 to 1 with an average mean factor congruence per solution (*M*_*mean*_) of .997, and an average minimum factor congruence per solution ($M_{\min \limits }$) of .98. After varimax rotation, the factor congruence ranged overall between −.16 and 1 (*M*_*mean*_ = .996, $M_{\min \limits } = .98$), and after promax rotation, it ranged overall between .10 and 1 (*M*_*mean*_ = .99, $M_{\min \limits } = .95$).

Despite the mostly small differences in loadings and pattern coefficients, the indicator-to-factor correspondences differed between the final promax-rotated R psych and SPSS solutions in 41.7% of all data sets and in 45.0% of the 200 data sets with a solution with two or more factors. This mostly concerned differences for 1 to 3 indicators, but it went up to differences for 21 indicators in the most extreme case. Finally, the mean and maximum absolute differences in pattern coefficients featured a credible association with the indicator-to-factor ratio in a Bayesian gamma regression analysis. A lower ratio was related to higher mean (*b* = − 0.13, 95% HDI[− 0.19; − 0.06], *r*_*s*_ = −.21) and maximum (*b* = − 0.15, 95% HDI[− 0.22; − 0.08], *r*_*s*_ = −.28) differences in pattern coefficients.

### Discussion

When comparing the R psych and SPSS factor solutions for real data sets, we found mostly small differences in loadings and pattern coefficients and mostly large factor congruences. Still, for 41.7% of data sets the differences in pattern coefficients were large enough to result in different indicator-to-factor correspondences. The larger differences for pattern coefficients after promax rotation suggest that differences in results are mostly due to the different implementations of promax rotation in R psych and SPSS (i.e., using an unnormalized versus normalized target matrix, respectively) and due to promax rotation amplifying pre-existing differences after varimax rotation, and less due to the different implementations of PAF and varimax rotation.

These results reveal that applying the (allegedly) same EFA procedure to the same data in the two programs can result in considerably different factor solutions. A researcher working with one program would thus often draw different conclusions concerning which indicator loads on which factor or even concerning the adequate number of factors to retain compared to a researcher working with the other program. In applications like scale construction, such differences might ultimately even lead to different scales.

It thus seems that the ways through the jingle jungle are indeed different enough to call for a guide towards the best way through. As these analyses were based on real data sets, we do not know the “true” population models behind them. It therefore remains unclear where these differences come from and whether there is an implementation that results in more accurate results in general. For example, certain properties of the data sets might influence differences in solutions between the implementations and the accuracy of results. The present results suggest that the indicator-to-factor ratio might be one such property. As a next step, we thus performed a simulation study comparing many different implementations of PAF and promax, including the R psych and SPSS implementation, to approach these questions systematically, and to find the best way through the jungle in the form of a recommendation about which implementation to use for most accurate results.

## Differences in accuracy between implementations for simulated data

To be able to compare different implementations of PAF and promax in terms of their accuracy, a true model is needed for comparison. To this end, we simulated data based on different population models implementing various data structures. We then examined how well different implementations, featuring all possible combinations of the above identified settings of PAF and promax rotation, would recover the true solutions. That is, the aim of this simulation study was to search the space of possible implementations, including the R psych and SPSS ones, to test whether we could identify one implementation that would reliably yield more accurate solutions and would thus be preferable overall or at least for certain data structures. In addition, we also directly compared the R psych and SPSS implementations in more detail regarding their ability to recover the population models, as well as in terms of differences in pattern coefficients, in a separate simulation analysis. We report this latter analysis in the SM (Section 3).

### Methods

To compare the implementations regarding their ability to recover the underlying population model, we created 27 different pattern matrices and four different factor intercorrelation matrices, the combination of which resulted in 108 population models (see Table 4 for an overview, and SM, Sections 5 and 6, for the pattern- and factor intercorrelation matrices). Therein, we varied (a) the number of factors, (b) the number of indicators per factor, (c) the size of the pattern coefficients, (d) whether cross-loadings were present, and (e) the magnitude of the factor intercorrelations. Some of these population models were based on De Winter and Dodou (2012), yet we also added additional ones to cover a large space of possible data structures. For example, intelligence tests often exhibit a relatively low indicator-to-factor ratio and strong factor intercorrelations (e.g., Frazier & Youngstrom, 2007). Other measures, such as some personality scales, tend to have higher indicator-to-factor ratios with lower factor intercorrelations (e.g., Goldberg, 1999; Johnson, 2014). Simulating a diverse set of data structures permitted us to compare the implementations not only in general, but also on more specific levels, conditional on the data structure.

From each of these population models, we simulated two times 1000 data sets from multivariate normal distributions with sample sizes of 180 and 450,^6^ respectively, for the model recovery. To this end, we used the following procedure to simulate each data set from a population model, which always consists of a combination of a population pattern matrix and a population factor intercorrelation matrix. We first obtained the population correlation matrix $\boldsymbol {\mathcal {R}}$ from the population pattern matrix **Λ** (see Table 4, and SM, Section 5) and population factor intercorrelation matrix **Φ** (see Table 4, and SM, Section 6) with

1 $$ \boldsymbol{\mathcal{R}} = \boldsymbol{{\Lambda}} \boldsymbol{{\Phi}} \boldsymbol{{\Lambda}}^{T} $$

2 $$ \mathit{diag} (\boldsymbol{\mathcal{R}}) = 1 $$

For simplicity, we set the variable means to zero and the SDs to 1, thus the population covariance matrix equals $\boldsymbol {\mathcal {R}}$. We then sampled data (*N* = 180 or *N* = 450) from a multivariate normal distribution based on the respective covariance matrix.

For each simulated data set, we conducted each of the different implementations of PAF with subsequent promax rotation by extracting the true number of factors of the population models (i.e., either three or six factors). Regarding the PAFs, we varied the following settings: Three initial communality estimates—unity, MAC, and SMCs; whether the absolute of the eigenvalues should be used or not; whether the convergence criterion is tested against the maximum difference in any single communality estimate, or against the difference in the sum of all communalities; and two different convergence criteria, namely 10^− 3^ and 10^− 6^. Regarding the promax rotation, we varied the following settings: the two varimax types; whether the target matrix is normalized or not; and two different *k* parameters, namely (a) 4 in all cases versus (b) 3 in the case of unnormalized promax and 2 in the case of normalized promax (see Tataryn et al., 1999). This resulted in 192 possible implementations which we compared against each other in terms of their ability to recover the underlying population model.

#### Statistical analyses

We compared the 192 different implementations in terms of the root mean squared errors (RMSE, i.e., the deviance of the fitted sample pattern matrix from the true population pattern matrix), the probability for the occurrence of Heywood cases (see definition above), and the number of incorrect indicator-to-factor correspondences (see definition above). The RMSE between the population pattern matrix **Λ** and a fitted sample pattern matrix $\boldsymbol {\hat {\Lambda }}$ was computed with

3 $$ \mathit{RMSE} = \sqrt{\frac{\mathit{trace} [(\boldsymbol{{\Lambda}} - \hat{\boldsymbol{\Lambda}})^{T} (\boldsymbol{{\Lambda}} - \hat{\boldsymbol{\Lambda}})]}{\mathit{pm}}} $$

where *p* is the number of indicators, and *m* is the number of extracted factors.

We performed the following analyses separately for each of the 216 different models (108 population models times the two sample sizes). Moreover, for each of these models, we analyzed simulated data sets for which any of the *m* largest eigenvalues were negative during the PAF procedure for any of the implementations separately from those where all *m* largest eigenvalues were always positive.

We first ordered the different implementations from best to worst (i.e., lowest to highest), according to the average RMSE (MRMSE), proportion of Heywood cases, and number of incorrect indicator-to-factor correspondences, respectively. Next, we compared the best and the second-best implementation with Bayesian regressions with a dummy coded identifier for the implementations as predictor. The regression model with the RMSE as dependent variable was implemented with a Gaussian family and an identity link function; the one with the probability of Heywood cases was implemented with a binomial family with a logistic link function; and the one concerning the number of incorrect indicator-to-factor correspondences was implemented with a negative binomial family with a log link function. All models were run using the *rstanarm* package (Goodrich et al., 2018) with default priors and results were inspected using the *bayestestR* package, version 0.7.2 (Makowski et al., 2019).

To test the credibility of effects, we employed the region of practical equivalence (ROPE) plus 95% HDI rule (Kruschke, 2018) for the linear and logistic regressions. That is, if the 95% HDI of a parameter fell completely outside the ROPE, we regarded this as conclusive evidence for an effect. If less than 95% of the HDI fell outside or inside the ROPE, we regarded this as inconclusive evidence for an effect. Finally, if the 95% HDI fell completely inside the ROPE, we regarded this as conclusive evidence that there was no effect. These analyses permitted us to judge whether one implementation was generally better suited for particular data structures. We defined the ROPE to be [− 0.1 ∗ *SD*_*dv*_,0.1 ∗ *SD*_*dv*_] for linear regression models and [− 0.18,0.18] for logistic regression models (in line with Makowski et al., 2019; Kruschke, 2018). Because there is no standard for choosing a ROPE for negative binomial regressions, we simply relied on the 95% HDI rule to gauge whether an effect was credible.

For analyses of data sets where all *m* largest eigenvalues were positive, regression analyses were only run if at least ten of the 1000 simulated data sets per population model resulted in all-positive *m* largest eigenvalues. Conversely, for analyses of data sets with some negative *m* largest eigenvalues, regression analyses were only run if at least ten of the 1000 simulated data sets per population model resulted in some negative *m* largest eigenvalues. Moreover, a logistic regression was only run if additionally at least 1% of the data sets of the current population model contained a Heywood case, and a negative binomial regression was only run if additionally there were at least two unique numbers of incorrect indicator-to-factor correspondences present. This was to ensure that the models could be run and did not result in errors (e.g., due to no variance in the dependent variable).

If there was conclusive evidence for a difference between the best and second-best implementation, the procedure was stopped and counted as conclusive evidence for a difference between settings in the respective population model. If the evidence was inconclusive or there was conclusive evidence for equality of the solutions found by the two implementations, the best solution was compared to the third-best solution, and so on, either until conclusive evidence for a difference was found or until all 191 other implementations were compared against the best one. If after this procedure there was conclusive evidence for equality between the best and the worst implementation, we counted this as conclusive evidence for the absence of relevant differences between the implementations in the respective population model. If there was conclusive evidence neither for a difference nor for equality, the evidence was counted as inconclusive. Moreover, if no regression models could be run (e.g., because not a single Heywood case occurred for any implementation in a population model), differences were gauged descriptively. In these cases, we counted a complete match between all implementations (e.g., when none of the implementations resulted in a Heywood case) as conclusive evidence for equality, whereas an imperfect match was counted as inconclusive evidence.

To analyze the simulation results, we first descriptively compared the different implementations regarding their MRMSE, proportion of Heywood cases, and incorrect indicator-to-factor correspondences. Moreover, to determine which implementation on average produced the best results, we used the proportion across the 108 population models with which a given implementation was among the best ones (i.e., was the best implementation, had conclusive evidence for equality with the best implementation, or had inconclusive evidence for a difference to the best implementation) as determined with the regression analyses above. We then computed a weighted mean (where the number of datasets entered were the weights) across these proportions to determine which implementations performed best overall.

### Results

Results from our regression analyses revealed conclusive evidence for differences between at least some of the 192 implementations for the majority of the population models regarding RMSE, and for nearly half of the population models regarding the correctness of indicator-to-factor correspondences (see Table 1). Regarding the proportion of Heywood cases, there was conclusive evidence for practical equivalence of the implementations. Although the differences in accuracies were mostly small, there were some population models—namely those with very low indicator-to-factor ratios as well as those with mixed or high factor intercorrelations—where differences were larger.

**Table 1:** Evidence for differences in accuracy, proportion of Heywood cases, and correctness of indicator-to-factor correspondences between implementations

	*N* = 180			*N* = 450		
Property	Inc	Eq	Diff	Inc	Eq	Diff
Data sets without negative eigenvalues						
RMSE	17	0	91	11	1	96
Heywood	0	108	0	0	108	0
Ind-to-Fac Corres	31	32	45	19	46	43
Data sets with negative eigenvalues						
RMSE	1	0	9	0	0	9
Heywood	0	10	0	0	9	0
Ind-to-Fac Corres	5	2	3	1	0	8

Tally of type of evidence for the 216 different models (108 population models × 2 sample sizes) derived from Bayesian regression analyses. The row sum for the rows concerning data sets with negative eigenvalues are smaller than 216 because data sets with negative eigenvalues occurred only for some models. RMSE = Root mean squared error. Heywood = Probability of the occurrence of a Heywood case. Ind-to-Fac Corres = Difference in indicator-to-factor correspondence from found solution to population model. Inc = Inconclusive evidence. Eq = Conclusive evidence for no relevant difference between the implementations (equality). Diff = Conclusive evidence for a difference between at least some implementations

Moreover, there was no single implementation that performed best across all conditions and regarding all three considered accuracy criteria. However, there were some settings that were clearly superior, namely sum for criterion type and a convergence criterion of 10^− 3^ (both in the PAF procedure). A description and comparison of the implementations that produced the best results in one of the considered criteria is included in Table 2 and, for a larger set of implementations, in Table *best_implementations.xlsx* in the online repository, https://osf.io/6prcz/).

**Table 2:** Proportion of population models for which the best implementation and the R psych and SPSS implementations were among the best

	Best	Psych_unity_	Psych_SMC_	SPSS
RMSE				
*N* = 180, pos. eigen.	.69	.46	.52	.67
*N* = 180, neg. eigen.	.50	.10	.00	.60
*N* = 450, pos. eigen.	**.72**	.45	.56	.68
*N* = 450, neg. eigen.	.78	.00	.00	.78
*WM*_*RMSE*_	.70	.45	.53	.68
Heywood cases				
*N* = 180, pos. eigen.	**1**	**1**	**1**	**1**
*N* = 180, neg. eigen.	**1**	**1**	.00	**1**
*N* = 450, pos. eigen.	**1**	**1**	**1**	**1**
*N* = 450, neg. eigen.	**1**	**1**	.00	**1**
*WM*_*Heywood*_	**1**	**1**	.98	**1**
Ind.-to-Fac. Corres.				
*N* = 180, pos. eigen.	.93	.70	.93	.91
*N* = 180, neg. eigen.	**1**	.70	.00	**1**
*N* = 450, pos. eigen.	.92	.72	.86	.90
*N* = 450, neg. eigen.	**.89**	.11	.00	**.89**
*WM*_*Ind*.*−to−Fac*.*Corres*._	**.93**	.70	.88	.91
*WM*_*overall*_	**.88**	.72	.80	.86
Settings				
PAF				
Communality method	SMC	unity	SMC	SMC
Criterion type	sum	sum	sum	max. ind.
Absolute eigenvalues	yes	no	no	yes
Convergence criterion	10^− 3^	10^− 3^	10^− 3^	10^− 3^
Promax rotation				
Varimax type	kaiser	svd	svd	kaiser
P type	norm	unnorm	unnorm	norm
*k*	4	4	4	4

For positive eigenvalues, the proportion of the 108 population models for which the respective setting combination was among the best setting combinations is shown. For negative eigenvalues, the proportion of the population models including data sets that resulted in negative eigenvalues for which the respective setting combination was among the best setting combinations is shown. The top row contains the identifiers of the implementations, their settings are listed in the bottom part of the table. Boldface indicates that this implementation was most frequently among the best implementations for the respective data sets. Best = implementations with best results overall; Psych_unity_/Psych_SMC_ = R psych implementation with unity/SMC as initial communality estimates; SPSS = SPSS implementation; RMSE = root mean square error; pos. eigen. = all-positive eigenvalues; neg. eigen. = some negative eigenvalues; *WM* = Weighted mean, where the weights are the number of datasets used in the respective regression analyses (those with negative eigenvalues made up only about 2% of all datasets and are thus weighted much less strongly; see Table *best_implementations.xlsx* in the online repository, https://osf.io/6prcz/); Ind.-to-Fac. Corres. = indicator-to-factor correspondences; PAF = principal axis factoring; P type = target matrix type; k = power in promax; MAC = maximum absolute correlation; SMC = squared multiple correlation; sum = deviance of the sum of all communalities; max. ind. = maximum absolute deviance of any communality; unnorm = unnormalized; norm = normalized; svd = singular value decomposition

Regarding RMSE, the implementations that produced the most accurate results differed for the sample sizes of 180 and 450. For PAF, the best implementations in the case of *N* = 180 all used MACs as initial communality estimates, sum as criterion type, and a convergence criterion of 10^− 3^ (both treatments of eigenvalues produced equally accurate results). For the promax rotation, they used an unnormalized target matrix with *k* = 3 (both varimax types produced equally accurate results). When the sample size was 450, SMCs were used as initial communality estimates instead of MACs, and only absolute eigenvalues were used—the other settings were the same. For the promax rotation, the best implementations then used a normalized target matrix with *k* = 4 and kaiser as varimax type (although the varimax type only had a very small influence). Regarding Heywood cases, there was conclusive evidence for equality between all implementations.

Finally, regarding indicator-to-factor correspondences, the best implementations for PAF were again ones that used sum as criterion type, a convergence criterion of 10^− 3^, either SMCs or MAC as initial communality estimates, as well as absolute eigenvalues. For promax, a normalized target matrix with *k* = 4 was best, with singular value decomposition as varimax type in combination with MACs, and kaiser as varimax type in combination with SMCs.

**Fig. 2:**
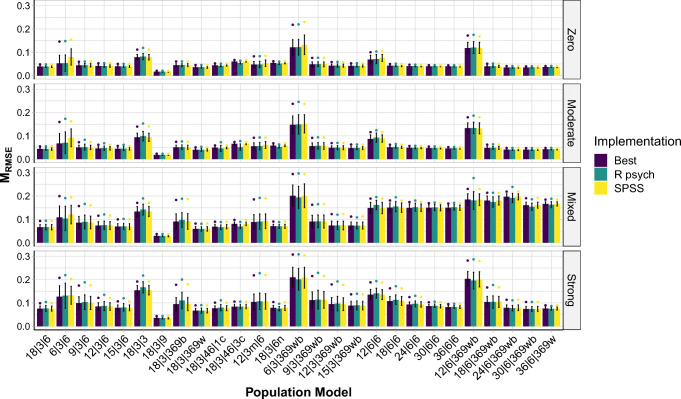
Distributions of RMSE of three different implementations, separately for the different population models, based on data sets simulated with *N* = 450. A population model is always a combination of a pattern matrix (on the *x*-axis) and a factor intercorrelation matrix (the facets). *Bars* indicate the mean RMSE, the *whiskers* indicate ± 1 *SD*, and *dots* represent the mean of the 5% largest RMSE. Best = implementation with best results overall; R psych = R psych implementation with SMC as initial communality estimates when no negative eigenvalues occurred and R psych implementation with unity as initial communality estimates when negative eigenvalues occurred; SPSS = SPSS implementation. See Table 2 for more information on these implementations. An overview of the population models is provided in Table 4. The detailed population pattern- and factor intercorrelation matrices are provided in the SM, Sections 5 and 6

On average, the impact of the different implementations, although in many cases statistically robust (see Table 1), tended to be smaller than that of the data structures—that is, the population models (see Fig. 2 and Fig. S6). To gauge the impact of the data structures, we computed the differences between the best- and worst-performing implementation in a given population model, separately for every population model.^7^ Conversely, to gauge the impact of the implementations, we computed the differences between the population models leading to the best and worst performance in a given implementation, separately for each implementation. In this analysis we focused on the data sets with *N* = 450.

**Fig. 3:**
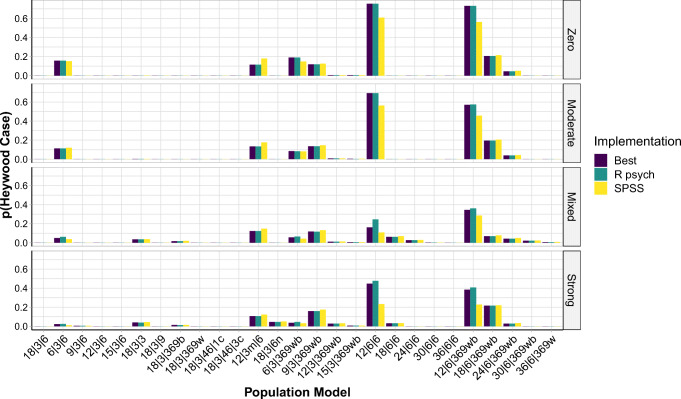
Proportions of solutions per implementation out of the 1000 simulated data sets in which Heywood cases occurred, separately for the different population models, based on data sets simulated with *N* = 450. A population model is always a combination of a pattern matrix (on the *x*-axis) and a factor intercorrelation matrix (the facets). Best = implementation with best results overall; R psych = R psych implementation with SMC as initial communality estimates when no negative eigenvalues occurred and R psych implementation with unity as initial communality estimates when negative eigenvalues occurred; SPSS = SPSS implementation. See Table 2 for more information on these implementations. An overview of the population models is provided in Table 4. The detailed population pattern- and factor intercorrelation matrices are provided in the SM, Sections 5 and 6

**Fig. 4:**
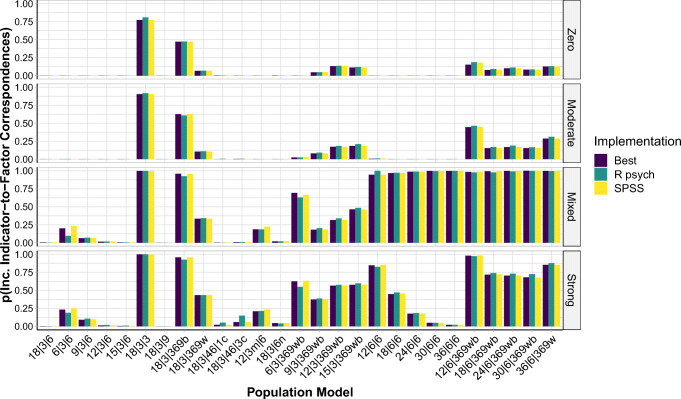
Proportions of solutions per implementation out of the 1000 simulated data sets with at least one incorrect indicator-to-factor correspondence, separately for the different population models, based on data sets simulated with *N* = 450. A population model is always a combination of a pattern matrix (on the *x*-axis) and a factor intercorrelation matrix (the facets). Best = implementation with best results overall; R psych = R psych implementation with SMC as initial communality estimates when no negative eigenvalues occurred and R psych implementation with unity as initial communality estimates when negative eigenvalues occurred; SPSS = SPSS implementation. See Table 2 for more information on these implementations. An overview of the population models is provided in Table 4. The detailed population pattern- and factor intercorrelation matrices are provided in the SM, Sections 5 and 6

Based on this approach, we found that across all implementations the differences in MRMSE ranged from .00 to .10 (*Mdn* = .01; *M* = .02). In contrast, across the population models, the differences in MRMSE ranged from .18 to .28 (*Mdn* = .21; *M* = .22). Moreover, the difference in the proportion of Heywood cases across all implementations ranged from .00 to .92 (*Mdn* = .00; *M* = .15), and the difference in proportion of Heywood cases across the population models ranged from .24 to .98 (*Mdn* = .87; *M* = .78; see also Fig. 3 and Fig. S7). Finally, across the implementations the differences in the proportion of solutions with at least one incorrect indicator-to-factor correspondence ranged from .00 to .53 (*Mdn* = .01; *M* = .06), while, across population models, this difference in proportions was always 1.00 (see also Fig. 4 and Figs. S8–S10). Overall, the data structures that led to larger errors and problems regarding Heywood cases and indicator-to-factor correspondences were ones with high and mixed factor intercorrelations, with low or heterogeneous pattern coefficients, and with low indicator-to-factor ratios.

### Discussion

Our simulations to compare many possible implementations of PAF and promax led to two main insights. First, we found that many statistically robust differences between implementations occurred and were able to pinpoint properties of implementations that led to the best results regarding RMSE and indicator-to-factor correspondences. The best trade-off seems to be to use SMCs as initial communality estimates, sum as criterion type, absolute eigenvalues, and a convergence criterion of 10^− 3^ for PAF; and a normalized target matrix with *k* = 4 for promax with kaiser as varimax type (although singular value decomposition as varimax type performs almost as well). This implementation (named *best* in Table 2) is available as default in the *EFAtools* R package (Steiner & Grieder, 2020), along with the possibility of varying the settings as done in this simulation study.

With smaller samples, using MACs instead of SMCs and an unnormalized target matrix with *k* = 3 could be beneficial to maximize the accuracy of the pattern coefficients. However, this comes at the cost of a higher probability for erroneous indicator-to-factor correspondences.

The second main insight is that the data structures had a large impact on the recovered factor solutions. For example, a low indicator-to-factor ratio often led to problems such as worse fit, the occurrence of Heywood cases, and problems in the identification of the correct indicator-to-factor correspondences. Moreover, solutions with weak factors also displayed worse fit. Finally, large factor intercorrelations and weak factors led to severe problems in recovering the correct indicator-to-factor correspondences, which might limit the robustness of EFA (or at least of PAF and promax rotation) for this kind of data structures.

To sum up, we succeeded in finding a best way through the jingle jungle in the form of the best implementation identified here. Another approach might be to incorporate the full jungle into one’s analyses by using model averaging. We further discuss this possibility below.

## General discussion

We compared an EFA procedure with PAF and promax rotation between the two most prominently used programs in psychological research (Dunn, 2011): R (using the popular *psych* package) and SPSS. We indeed discovered a jingle jungle for the investigated EFA procedure and the programs considered: Equal names do not mean equal implementations, and with this do not necessarily mean comparable results. But how different are the results? And is there a best way through the jungle?

Our main findings can be summarized in three points. First, we found many systematic differences in pattern coefficients and in accuracy between the tested implementations, including the R psych and SPSS implementations. Although most differences in (the accuracy of) pattern coefficients were small, very large ones occurred for some data sets and population models, and many led to differences in (the accuracy of) indicator-to-factor correspondences. Second, neither of the two implementations—R psych or SPSS—consistently resulted in more accurate solutions than the other across all population models. The implementations producing the most accurate results for PAF and promax rotation are combinations of the R psych and SPSS implementations. Third, the data structure is at least as important as the implementation for the accuracy of results. For some data structures, the accuracy is very low, regardless of the implementation.

### Mostly small, but systematic differences

As is evident from results from both the real data analysis and the simulation study, the different implementations—including R psych and SPSS—will lead to comparable results in many cases. Nevertheless, we found pattern coefficients differed systematically between implementations, with conclusive evidence for differences in accuracy for 87% of the population models. Moreover, for the real data sets, the average maximum difference between the R psych and SPSS solutions was a non-negligible 0.12. Perhaps even more importantly, the indicator-to-factor correspondences differed between R psych and SPSS for 42% of the real data sets, and their accuracy differed between implementations for 42% of the population models in our simulation study as well. Due to the use of thresholds, even small differences in pattern coefficients might affect indicator-to-factor correspondences and with this even decisions on which solution to retain. A strict use of thresholds is of course questionable. Nevertheless, thresholds are applied and they may be used to too strongly defend a more or less arbitrary choice about which solution to retain, especially if this solution is in accordance with a favored theory.

Overall, our results demonstrate that different implementations of PAF and promax rotation—with promax rotation likely having the greater impact—can lead to fairly different solutions, and in many cases also to different conclusions regarding the factor structure of the investigated construct. Given the amount and impact of these differences, a natural question to ask is whether there is one implementation of PAF and promax rotation that renders the most accurate results under different conditions.

### Best PAF and promax implementations

As stated above, none of the considered implementations—including R psych and SPSS—consistently outperformed all others. Yet, our simulation analyses still permitted us to identify specific settings that seem to be advantageous and allowed us to make a general recommendation for implementations of PAF and promax rotation. These implementations constitute a combination of the R psych and SPSS implementations. Specifically, for PAF, one should take the absolute eigenvalues (as SPSS does), compute initial communalities with SMCs, use 10^− 3^ as convergence criterion, and apply the convergence criterion to the sum of all communalities (as R psych does). For promax rotation, the varimax type *kaiser* should be used, and a row-normalization should be done on the target matrix of the varimax solution (as done in SPSS) and *k* = 4 should be used as the power to which to raise the elements of the target matrix. These combinations of settings are implemented as default for PAF and promax rotation in the *EFAtools* package.

Although this implementation performed best on average, our results also reveal that the choice of one implementation over the other will probably have a major impact on results only in certain cases. Our recommended implementation will therefore maximize the verisimilitude probably especially for more “problematic” data structures like the ones discussed in the next paragraph.

### Data structure is at least as important as implementation

Our simulation analyses revealed that the data structure had a strong influence on the accuracy of a solution. For cases with a low indicator-to-factor ratio, weak factors, and large factor intercorrelations (independent of each other), the accuracy of results was often very limited, to the point that, for some cases, nearly all of the 1000 solutions had at least one incorrect indicator-to-factor correspondence (see Fig. 4).

The finding that a low indicator-to-factor ratio can be problematic in EFA is in line with previous research (e.g., MacCallum et al., 1999). Specifically, a low indicator-to-factor ratio results in less stable and less replicable factor solutions (MacCallum et al., 1999; Tucker & MacCallum, 1997; Gorsuch, 1983; Mulaik, 2010). Regarding factor intercorrelations, it has been shown that a positive manifold (i.e., exclusively positive intercorrelations) leads to better recovery of factor solutions (Tucker & MacCallum, 1997). Our results somewhat contradict these findings: In our simulation study, factor structures with mixed (.30, .50, .70) and with high (.70) factor intercorrelations resulted in a less accurate recovery of the true factor solutions, while orthogonal factors caused the least problems. This is true for both investigated sample sizes. However, others have also proclaimed (Gorsuch, 1983) and shown (Gerbing & Hamilton, 1996) that too high factor intercorrelations can be problematic in factor analysis, especially for the recovery of weak factor loadings (De Winter & Dodou, 2012). These findings are corroborated by our analyses, where factors with low pattern coefficients (.30) were also worse recovered compared to factors with higher pattern coefficients (.60 or .90), especially if factor intercorrelations were high. Previous studies focusing exclusively on orthogonal factor structures have also demonstrated worse recovery of weak compared to stronger factors (MacCallum et al., 1999; Hogarty, Hines, Kromrey, Ferron, & Mumford, 2005). Finally, the data structure also influences the sample size necessary for a stable factor solution. That is, a stable solution can be achieved with smaller samples if the indicator-to-factor ratio is high, if communalities are high (strong factors), and if the factors are correlated (Tucker & MacCallum, 1997; MacCallum et al., 1999; Hogarty et al., 2005).

Despite these findings, problematic data structures, such as a low indicator-to-factor ratio, became increasingly common in factor-analytic research, especially on intelligence tests (Frazier & Youngstrom, 2007) and are still quite common to date (Goretzko, Pham, & Bühner, 2019). It is therefore important that simulation studies in a factor-analytic framework take these problematic data structures into account.

### Model averaging: An alternative way through the jungle?

Given that the variation in factor solutions across implementations was larger for problematic data structures, the amount of variability between different implementations might be useful to judge the stability of the factor solution for a particular data set. Thus, applying multiple implementations of the same procedure, and possibly also different extraction and rotation methods,^8^ could serve as a robustness check for a factor structure. A larger variation in the factor solutions across the different implementations or methods would indicate a more unstable factor structure for this particular data set and could render it more likely that the data structure is problematic. Researchers could then use this as a guide to judge whether more indicators or more participants should be sampled.

Applying multiple implementations or methods with a common purpose also allows to calculate an average factor solution across all different implementations and methods, which could be preferable to results from a single implementation as it takes model uncertainty into account. With this, we get in the realm of model averaging, which Fletcher (2018) defines as “a means of allowing for model uncertainty in estimation which can provide better estimates and more reliable confidence intervals than model selection” (p. 1). It typically involves calculating a weighted average of the model parameters, with stacking as the preferred method of weighting for frequentist model averaging, and another common method being AIC weights (Fletcher, 2018). Model averaging has mainly been used in regression frameworks and on models with different sets of predictor variables (Schomaker and Heumann, 2011; Fletcher, 2018; Steel, 2020). For EFA, there are some studies using averaging to get more robust estimates (e.g., Gerbing & Hamilton, 1996 who averaged across different rotation methods), and one study used model averaging with AIC weights across ML estimated solutions with different numbers of factors (Schomaker & Heumann, 2011). To our knowledge, however, there is no study that investigated model averaging across different settings or methods within the EFA framework.

In sum, model averaging might be a possibility not only to provide a straight path through the jingle jungle ignoring the surroundings, like our identified best implementation, but rather to produce a full map of the jungle, and possibly also an even better way out of it in the form of an average factor solution. For an easy application and first step towards model averaging we include a function in the *EFAtools* package to flexibly perform and average across different implementations of PAF, varimax, and promax rotation, as well as different extraction and rotation methods with unit-weighting. Clearly, future research is needed to investigate how model averaging is best implemented for EFA to maybe result in more stable and reliable parameter estimates compared to using a single EFA model. One challenge in this will be to identify which weights to use for averaging. For PAF, AICs cannot be used sensibly, and stacking is not practicable for EFA, either. Until these questions are answered, this kind of model averaging may mainly be useful for testing the robustness of a factor solutions across different implementations, and researchers may want to rely on the best implementation identified here as a best guess for the model parameters.

### Implications

One implication of our study is that researchers should not assume that procedures with the same name are implemented the same way and necessarily lead to comparable results. A consequence of such incomparable results from factor-analytical methods is that different researchers might draw different conclusions about the latent structure of their data, depending on which program they used. This could be especially problematic if EFA is used to create new instruments or as a tool in theory building. Moreover, it could lead to results appearing not to be replicable as a consequence and as such might even contribute to the replication crisis (see Ershova & Schneider, 2018; Flake et al., 2017). If different programs are thought to lead to comparable results, differences in implementations might not be considered as an explanation for the failure to replicate an analysis. This bears the potential for waste of money and time to find conceptual or methodological explanations for differences that might actually just be an artifact of the procedure used.

To counteract possible misconceptions and to facilitate comparisons of implementations of the kind performed here, we advocate the use of free and open-source software (e.g., R, Python, or Julia) and the sharing of analysis scripts. Doing so empowers other researchers to track the analyses and, if questions arise, to dig into the code of the actual procedures. Such comparisons are harder to do with proprietary software as one needs to have the appropriate license and even then one has to rely on reconstructions of the procedures in most cases because the source-code is not publicly available. Another advantage of the use of open-source software is that newly developed or enhanced procedures can be shared immediately.

Our results also demonstrate that researchers should be cautious when interpreting their EFA results. They might be overconfident in their factor-analytical results and what these tell them about the “real” structure of the assumed underlying latent construct (see Yarkoni, 2020). However, especially for data structures like those often present in intelligence tests (low indicator-to-factor ratio, high factor intercorrelations, and weak first-order factors), EFA seems to have difficulties recovering the data structure, even when there exists a “true” underlying model and data are simulated from multivariate normal distributions, as in our simulations. Future studies could vary more properties of both real and simulated data sets. For example, it would be interesting to see how the presence of ordinal data or departures from (multivariate) normality, in combination with different correlation methods, might influence results. Moreover, the poor recovery of solutions with high factor intercorrelations could be a problem for oblique rotation methods in general, not only for promax rotation. Future studies should therefore compare the performance of different rotation methods in the recovery of such data structures.

Given these issues, researchers might be tempted to attribute these to the exploratory nature of EFA and to instead put their trust in (presumably) less data-driven methods, such as confirmatory factor analysis (CFA). However, the same kind of data structure that poses difficulties to EFA is problematic for CFA as well (e.g., Marsh, Hau, Balla, & Grayson, 1998; Ximénez, 2006, 2009). This is not surprising, given that EFA and CFA are not that different after all and neither is necessarily more data- or more theory-driven than the other (Schmitt, 2011).

Researchers intending to use factor-analytic methods should therefore design their tests and questionnaires such that the data are suitable for factor analysis; that is, ensure a large enough indicator-to-factor ratio (probably at least 5:1 or 6:1; e.g., Goretzko et al., 2019; Gorsuch, 1983) and a sufficiently large sample size (at least 400) that should be larger the smaller the indicator-to-factor ratio is, and if weak factors are expected, especially when paired with high expected factor intercorrelations (Goretzko et al., 2019; Gorsuch, 1983; Tucker & MacCallum, 1997; MacCallum et al., 1999; Hogarty et al., 2005; De Winter & Dodou, 2012). Thus, ensuring an appropriate data structure is a prerequisite for a stable and replicable factor solution. Last but not least, a factor structure identified with factor-analytic methods should always be tested against external criteria to ensure its validity.

### Limitations

One limitation of our work is that we only investigated a specific procedure within one statistical framework—one extraction method and one rotation method out of many, even though they are among the most prominent ones within EFA. But even within this narrow selection of procedures we found several differences in the implementations and the resulting solutions. Future research could investigate to what extent these issues apply to other factor-analytical methods (e.g., ML estimation) and statistical procedures. Similar jingle jungles have already been found between R and SPSS, for example, for linear regression (krissen, 2018; u/kriesniem, 2018), cox regression (GaryStats, 2017), multinomial logistic regression (Collins, 2016), and ANOVA (del Rio, 2017). Our guess is that many further jingle jungles might be found for other programs and statistical procedures. In fact, similar jungles have already been discovered for nonparametric statistical procedures across the four programs SPSS, SAS, Stata, and R, where the authors also point to the consequences of such variations for replication attempts (Hodges et al., 2020).

We could also only compare a selection of the various programs available to perform these analyses. As we chose the programs that are probably the most used in psychological research (Dunn, 2011), and as the psych package has also influenced the implementation of the EFA framework in Python (Biggs, 2019), our findings are likely relevant for many researchers performing EFA. Moreover, we also went beyond the implementations of these two programs and included all possible combinations of the identified settings for PAF and promax in our simulation analysis.

Another limitation is that we did not have access to the source code of SPSS and were therefore only able to reproduce its implementations by implementing mathematical formulas from the algorithms manual (IBM Corp, 2017) and comparing the original output from SPSS with the one from our reconstruction of the SPSS implementation. Especially for the varimax implementation, it was difficult to find out how exactly the procedure is implemented in SPSS, as results with the implementation based on the formulas in the algorithms manual were not comparable enough to the original SPSS results. After some adjustments to the varimax criterion, we managed a closer reproduction of original SPSS results. However, without access to the source code, it was impossible for us to determine the exact deviations of the implementation from the formulas provided in the algorithms manual. Nevertheless, we were able to reproduce results from SPSS with high accuracy and therefore believe that we reconstructed its algorithms well enough for the purpose of our analyses.

Finally, as is always the case for simulation studies, it is unclear how results from our simulation analyses generalize to other data structures and sample sizes not simulated here. However, we varied many different characteristics of the data structures which reflect many data structures occurring in EFA research (e.g., Goretzko et al., 2019). Similarly, it is unclear to what extent results from simulation studies generalize to real data analyses, where there is no true model underlying the data, “everything is correlated with everything” (Meehl, 1990, p. 123), and thus an infinitely large number of constructs may influence a correlational structure (Preacher, Zhang, Kim, & Mels, 2013; Meehl, 1990; MacCallum, 2003; Cudeck & Henly, 1991). This caveat is one reason why we also analyzed real data sets. The agreement of the results from our real data analyses, where we included a large number of diverse data sets, and from the simulation study might at least indicate some generalizability of results from our simulation study to real-world problems.

### Conclusions

Our results show that a jingle fallacy is indeed apparent in the investigated EFA procedure. That is, EFA methods named the same are actually implemented differently in the programs considered. The jingle jungle we discovered does have important implications, with different implementations frequently leading to different conclusions regarding the factor structure. We advocate the search for and exploration of further jingle jungles to gauge the extent of this issue for other statistical procedures and to enable researchers to make an informed decision about which program or implementation to use best. Moreover, we encourage researchers to state which version of a program they used for a particular analysis—as details in the implementations might be subject to change over time—as well as to familiarize themselves with default values provided in software packages to understand which specifications are used in their analyses. With the present work, we hope to raise awareness of possible differences in implementations of statistical procedures in different programs, and we advise researchers to use our recommended settings for PAF and promax rotation as the best way through the jungle, at least until other, potentially better ways through it—such as model averaging—have been tested sufficiently.

## Appendix

**Table 3:** Differences between the R psych and SPSS implementations

Procedure	Setting	R psych	SPSS	Note
PAF	Communality method	SMC, if this fails: unity	SMC, if this fails: MAC, if this fails, unity	How the diagonal of the original matrix is replaced to find initial eigenvalues
	Absolute eigenvalues	No	Yes	To avoid negative eigenvalues, SPSS takes the absolute of initial eigenvalues. This is not done in R psych, where negative eigenvalues might render the use of SMCs impossible
	Criterion type	Difference in sum of communalities	Difference in maximum individual communalities	Value on which the convergence criterion is applied
Promax	Varimax type	Singular value decomposition	Kaiser	SPSS follows the original varimax procedure from (Kaiser, 1958); likely with small changes in the varimax criterion), while R uses singular value decomposition
	Normalization of target matrix	Unnormalized	Normalized	The target matrix is row-normalized in SPSS, but not in R psych. This is not the Kaiser normalization, which is done in both implementations

A detailed description of the implementations of R psych and SPSS can be found in the supplemental material. PAF = principal axis factoring; SMC = squared multiple correlation; MAC = maximum absolute correlation, unity = all 1’s

**Table 4:** Description of population models used for simulation analyses

Pattern matrices	Number of factors	Number of indicators per factor	Size of pattern coefficients	Notes
a) Pattern matrices				
Case 18|3|6	3	6	.60	Same baseline model as used in (De Winter & Dodou, 2012)
Case 6|3|6	3	2	.60	
Case 9|3|6	3	3	.60	Case 5 in (De Winter & Dodou, 2012)
Case 12|3|6	3	4	.60	
Case 15|3|6	3	5	.60	
Case 18|3|3	3	6	.30	
Case 18|3|9	3	6	.90	
Case 18|3|369b	3	6	.30, .60, .90	Different pattern coefficients between factors. Case 7 in (De Winter & Dodou, 2012)
Case 18|3|369w	3	6	.30, .60, .90	Different pattern coefficients within factors (each factor two each). Similar to cases 8/9 in (De Winter & Dodou, 2012)
Case 18|3|46|1c	3	6	.60	One cross-loading of .40. Similar to case 10 in (De Winter & Dodou, 2012)
Pattern matrices	Number of factors	Number of indicators per factor	Size of pattern coefficients	Notes
Case 18|3|46|3c	3	6	.60	Three cross-loadings of .40 (One factor with 2 and one with 1 cross-loading). Similar to case 10 in (De Winter & Dodou, 2012)
Case 12|3m|6	3	2, 4, 6	.60	Similar to cases 11/ 12 in (De Winter & Dodou, 2012)
Case 18|3|6n	3	6	.60	Random variation in pattern coefficients added, drawn from a uniform distribution [-.2, .2]. Case 13 in (De Winter & Dodou, 2012)
Case 6|3|369wb	3	2	.30, .60, .90	Different pattern coefficients within one of the factors
Case 9|3|369wb	3	3	.30, .60, .90	Different pattern coefficients within and between factors
Case 12|3|369wb	3	4	.30, .60, .90	Different pattern coefficients within and between factors
Case 15|3|369wb	3	5	.30, .60, .90	Different pattern coefficients within and between factors
Case 12|6|6	6	2	.60	
Case 18|6|6	6	3	.60	
Case 24|6|6	6	4	.60	
Case 30|6|6	6	5	.60	
Case 36|6|6	6	6	.60	
Case 12|6|369wb	6	2	.30, .60, .90	Different pattern coefficients within and between factors
Case 18|6|369wb	6	3	.30, .60, .90	Different pattern coefficients within and between factors
Case 24|6|369wb	6	4	.30, .60, .90	Different pattern coefficients within and between factors
Case 30|6|369wb	6	5	.30, .60, .90	Different pattern coefficients within and between factors
Case 36|6|369w	6	6	.30, .60, .90	Different pattern coefficients within factors
Factor Inter- correlations	Size of Inter- correlations	Notes		
b) Factor intercorrelations				
Zero	.00	Same intercorrelations as used in (De Winter & Dodou, 2012)		
Moderate	.30			
Mixed	.30, .50, .70			
Strong	.70	Same intercorrelations as used in (De Winter & Dodou, 2012)		

A population model is always a combination of a population pattern matrix and a population factor intercorrelation matrix. All population models are available in the *EFAtools* package

## Footnotes

1 Such changes are often listed in a changelog or news file, but this still requires users to actively consult the changelog after every software update.

2 Communality refers to the proportion of variance of an indicator that can be accounted for by the common factors (Gorsuch, 1983) and thus provides an indication of how strongly the indicator is related to all other considered indicators.

3 Our results are also valid for SPSS version 23, and probably versions 24 to 26, as the algorithms for the investigated procedures did not change from version 23 through 27 according to the algorithms manuals (IBM Corp, 2014, 2016, 2017) and we found results to be identical between versions 23 and 27 for these procedures.

4 This factor retention method is available in both R (e.g., in the packages *EFAtools*; *psych*; *nFactors*, Raiche & Magis, 2020; *paran*, Dinno, 2018; and *hornpa*, Huang, 2015) and SPSS (with syntax provided by O’Connor (2000), updated version available at https://people.ok.ubc.ca/brioconn/nfactors/nfactors.html).

5 These admissibility criteria were administered automatically. Due to the large number of data sets, it was not possible to manually inspect the factor solutions for plausibility, as is usually done in EFA. The relatively low salience threshold of .2 (as opposed to the often-used .3) was used to acknowledge sampling error. Specifically because this threshold was also used to define indicator-to-factor correspondences and the lowest true pattern coefficients in the simulated population models were .3.

6 These sample sizes correspond to a ratio of sample size (*N*) to number of indicators (*p*) of 10 and 25 for our baseline model (18|3|6), respectively. According to (MacCallum et al., 1999), a sample size of 180 is rather small for conditions like the ones in our baseline model. The smaller sample size and *N:p* ratio corresponds with the median values for our real data sets (see Table *Real_data_description.xlsx*, https://osf.io/pcrqu/). Conversely, a sample size of 450 should be sufficient also for more “problematic” data structures like those with low indicator-to-factor ratios and weak factors, as we simulated in some of our population models.

7 Note that therefore not one single implementation was used as best for every population model, but the respective implementation that in the current population model, across the 1000 simulated data sets, resulted in the lowest MRMSE, proportion of Heywood cases, or proportion of solutions with at least one incorrect indicator-to-factor correspondence.

8 For rotation methods this would, of course, only make sense for the same type of rotation; that is, either multiple orthogonal methods or multiple oblique methods.
